# Effectiveness of a Web-Based Simulation in Improving Nurses’ Workplace Practice With Deteriorating Ward Patients: A Pre- and Postintervention Study

**DOI:** 10.2196/jmir.5294

**Published:** 2016-02-19

**Authors:** Sok Ying Liaw, Lai Fun Wong, Eunice Ya Ping Lim, Sophia Bee Leng Ang, Sandhya Mujumdar, Jasmine Tze Yin Ho, Siti Zubaidah Mordiffi, Emily Neo Kim Ang

**Affiliations:** ^1^National University of SingaporeSingaporeSingapore; ^2^National University HospitalSingaporeSingapore

**Keywords:** Web-based simulation, clinical deterioration, nursing education, online learning, transfer of learning, nursing practice

## Abstract

**Background:**

Nurses play an important role in detecting patients with clinical deterioration. However, the problem of nurses failing to trigger deteriorating ward patients still persists despite the implementation of a patient safety initiative, the Rapid Response System. A Web-based simulation was developed to enhance nurses’ role in recognizing and responding to deteriorating patients. While studies have evaluated the effectiveness of the Web-based simulation on nurses’ clinical performance in a simulated environment, no study has examined its impact on nurses’ actual practice in the clinical setting.

**Objective:**

The objective of this study was to evaluate the impact of Web-based simulation on nurses' recognition of and response to deteriorating patients in clinical settings. The outcomes were measured across all levels of Kirkpatrick’s 4-level evaluation model with clinical outcome on triggering rates of deteriorating patients as the primary outcome measure.

**Methods:**

A before-and-after study was conducted on two general wards at an acute care tertiary hospital over a 14-month period. All nurses from the two study wards who undertook the Web-based simulation as part of their continuing nursing education were invited to complete questionnaires at various time points to measure their motivational reaction, knowledge, and perceived transfer of learning. Clinical records on cases triggered by ward nurses from the two study wards were evaluated for frequency and types of triggers over a period of 6 months pre- and 6 months postintervention.

**Results:**

The number of deteriorating patients triggered by ward nurses in a medical general ward increased significantly (*P*<.001) from pre- (84/937, 8.96%) to postintervention (91/624, 14.58%). The nurses reported positively on the transfer of learning (mean 3.89, SD 0.49) from the Web-based simulation to clinical practice. A significant increase (*P*<.001) on knowledge posttest score from pretest score was also reported. The nurses also perceived positively their motivation (mean 3.78, SD 0.56) to engage in the Web-based simulation.

**Conclusions:**

This study provides evidence on the effectiveness of Web-based simulation in improving nursing practice when recognizing and responding to deteriorating patients. This educational tool could be implemented by nurse educators worldwide to address the educational needs of a large group of hospital nurses responsible for patients in clinical deterioration.

## Introduction

In contemporary acute health care settings, there have been increasing numbers of patients with complex health problems who are more likely to deteriorate during their hospital admission, leading to significant adverse events. This trend is expected to continue as a result of an increasing number of older and more acutely ill patients being cared for in general wards [1,2]. Adverse events are defined as unintended complications and injuries that lead to mortality, unplanned intensive care unit admissions, and cardiopulmonary arrests [3]. There is evidence suggesting that adverse events occurring in general wards are often related to suboptimal care provided by ward nurses [4]. Having the most frequent direct contact and responsibility for monitoring patient vital signs, ward nurses are in the best position to recognize warning signs, report these signs to appropriate health care staff, and initiate immediate actions before the arrival of appropriate help [5,6].

A patient safety initiative, the Rapid Response System (RRS), was widely implemented in acute care hospitals to improve the care of patients with unexpected clinical deterioration, with the aim of preventing serious adverse events [7]. This system consists of an afferent limb and an efferent limb. The afferent limb involves detecting patient deterioration and triggering a response, followed by the efferent limb where the response team treats and prevents further deterioration of the patient [8]. The problem of the afferent limb where ward nurses continue to fail in triggering patient deterioration still persists despite the implementation of track-and-trigger systems such as the Early Warning Score System (EWSS) [9,10].

Staff education is advocated for improving ward nurses’ competencies in the care of deteriorating patients [5,11,12]. To date, simulation using mannequins has been widely implemented to provide opportunities for nurses to problem solve clinical situations of patient deterioration in a safe and controlled environment [13,14]. However, given the resource-intensive nature of this simulation, which requires simulation facilities, trained facilitators, and small group learning, it has constraints in providing a scalable and sustainable training [15]. With the advancement in multimedia technology, these constraints could be eased as it is now possible for situating simulations in Web-based learning, known as Web-based simulation [16].

Web-based simulation has been widely adopted for training health professionals [16,17]. A Web-based simulation using a virtual patient, known as e-RAPIDS (Rescuing a Patient in Deteriorating Situations), was developed at the National University of Singapore (NUS) by a multidisciplinary health care team from academic and clinical institutions for undergraduate nursing training to enhance student nurses’ clinical performance in assessing and managing deteriorating patients. Using a randomized controlled trial (RCT) study, e-RAPIDS was shown to be at least as effective as, if not better than, the mannequin-based simulation in improving nursing performance [18]. The successful implementation of the Web-based simulation for undergraduate nurses has prompted us to further develop Web-based simulation for training of hospital nurses. As a large group of hospital ward nurses was to be trained, Web-based simulation appeared to be a more cost-effective training method compared to the mannequin-based simulation. To address the educational needs of hospital nurses, more learning content was added and delivered through a variety of instructional strategies (animation and multimedia instructional materials) in e-RAPIDS. The learning materials were validated by a hospital nurse educator and medical clinician [19]. The effectiveness of e-RAPIDS in improving hospital nurses’ competencies at assessing and managing clinical deterioration was demonstrated in a simulated environment through an RCT [20].

Although previous studies have used rigorous research methodologies like RCTs to evaluate the outcomes of e-RAPIDS [18,20], the quality of evidence was limited in the context of a simulated environment and at Level 2 (learning outcomes) of Kirkpartick’s hierarchy of educational outcomes [21]. This study aimed to evaluate the impact of e-RAPIDS in clinical settings on improving nursing practice in recognizing and responding to deteriorating patients. The outcomes were measured across the levels of Kirpatrick’s evaluation model adapted by Tochel et al [22] with changes in nursing practice on triggering rates of deteriorating patients (Level 4A) as the primary outcome measure. We hypothesized that the number of cases triggered by the nurses would increase after the e-RAPIDS training. Secondary outcome measures included motivation (Level 1), knowledge (Level 2b), and perceived training transfer (Level 3).

## Methods

### Study Designs, Setting, and Participants

After receiving approval from an institutional review board, a pre- and postintervention study was conducted on one surgical ward and one medical general ward at an acute care tertiary hospital. The hospital is a 991-bed teaching hospital at a university in Singapore. These two study wards were chosen for their specialties and their high triggering rates based on previously recorded trigger forms. All registered nurses (RNs) and enrolled nurses (ENs) who were working in these two wards during the study period were scheduled to undertake the e-RAPIDS training, as part of their continuous nursing education, at the Centre for Healthcare Simulation from June 1-August 14, 2014.

### Implementation of e-RAPIDS

**Figure 1:**
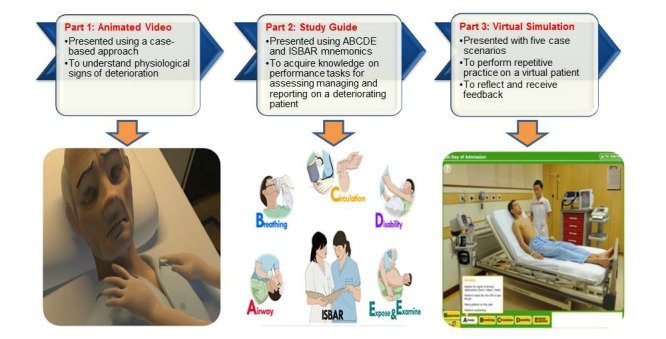
Learning path in e-RAPIDS.

The nurses were individually brought into a room equipped with a computer set-up. The RNs and ENs were assigned a different version of e-RAPIDS: the RN version for RNs and the EN version for ENs. They were all instructed to follow the learning path and complete three parts of the program (see Figure 1). The first part required the participants to watch an animated video that focuses on early detection of changes in vital signs. Using a case scenario of a deteriorating patient, the animated video presented the early underlying compensatory mechanisms that highlighted the importance of recognizing an increase in respiratory rate and heart rate as early signs of patient deterioration. The second part was a study guide, which was presented using multimedia instructional materials (eg, texts, illustration, and audio of lung sounds), on the list of performance tasks to assess, manage, and report on a deteriorating patient. These tasks were organized using the ABCDE (Airway, Breathing, Circulation, Disability, and Expose/Examine) and ISBAR (Identity, Situation, Background, Assessment, and Recommendation) mnemonics. The lists of tasks included in the RN version and EN version were based on the scope of practice and roles as RNs or ENs. The final part of the program was a virtual simulation that was embedded with five simulation scenarios associated with a patient who has deteriorating conditions. The nurses were instructed to attempt all five scenarios. In each scenario, the nurses were taken through 4 steps: (1) select appropriate actions from the control menus (eg, ABCDE and ISBAR) to assess, manage, and report on the deteriorating patient, (2) reflect on the simulation experience using the debriefing questions, (3) review the evaluation checklist and receive feedback on the actions taken in the simulation scenario, and (4) to undertake a short multiple-choice questionnaire (MCQ) to evaluate their knowledge of the subject content. The entire e-RAPIDS lasted approximately 2.5-3 hours, and the website can be accessed by anyone [23].

### Evaluation of e-RAPIDS

The Kirpatrick’s evaluation model, adapted by Tochel et al for classifying the outcomes of educational intervention, was applied in this study to guide the evaluation of e-RAPIDS. These levels include Level 1 (participation), Level 2a (modification of attitudes), Level 2b (modification of knowledge or skills), Level 3 (behavioral change), Level 4a (change in organizational practice), and Level 4b (benefit to patients/clients) [22].

#### Participation: Motivational Reaction (Level 1)

All participants were invited to complete the Instructional Material Motivation Survey (IMMS), which used a 5-point Likert scale, immediately after completing the e-RAPIDS. The survey assessed their motivational reactions to the program using the four characteristics (Attention, Relevance, Confidence, and Satisfaction) from Keller’s model of motivational design [24]. Cronbach alpha reported for this study was .79 for scores from all IMMS dimensions.

#### Modification of Knowledge (Level 2b)

The participants’ knowledge on clinical deterioration was assessed through the 30-item MCQ that was administered immediately before and after e-RAPIDS. Two sets of questionnaires, RN-MCQ for RNs and EN-MCQ for ENs, were developed that aligned with the program’s learning objectives. The content validity of the questionnaires was established by a panel of medical and surgical care experts.

#### Behavioral Changes: Training Transfer at Workplace (Level 3)

This level was evaluated through a self-reported questionnaire that was conducted 3-4 months after the e-RAPIDS took place. All nurses who had undertaken the learning were invited to complete a questionnaire, which used a 5-point Likert response scale, on their perceived training transfer at workplace. The questionnaire was adapted and modified from a previous study [25] to fit our study’s situation. While a previous study reported a Cronbach alpha of <.80 [25], our study obtained a high internal consistency of Cronbach alpha=.94.

#### Change in Organizational Practice: Trigger Cases (Level 4a)

Clinical records on cases triggered by ward nurses from the two study wards were checked by an investigator for frequency and types of triggers over a period of 6 months pre- (December 2013 to May 2014) and 6 months postintervention (August 2014 to February 2015). The types of trigger included respiratory rate, oxygen saturation, pulse rate, blood pressure, acute change in mental status, and serious concern. The patient characteristics of the two wards, including the number of admitted patients, number of occupied beds, length of stays, and age were also measured from the hospital admission.

### Data Analysis

Descriptive statistics of the study population and ward characteristics are presented using means, standard deviations, counts, and percentages. Means and standard deviation were calculated to examine the participants’ motivational reaction and perceived transfer of learning. Paired *t* test was used to examine any significant changes between the baseline and posttest knowledge scores. Independent sample *t* test was used to determine any significant differences between the RN and EN groups. Chi-square test or Fisher’s exact test was performed for comparisons of binomial proportions between the pre-and postintervention periods.

## Results

### Demographics

A total of 99 nurses (85% participation rate) from the surgical ward (53/99, 54%) and medical ward (46/99, 46%) participated in the e-RAPIDS training, with a total of 64 RNs and 35 ENs. Most of them were female (95/99, 96%) with an average age of 27.63 years (SD 5.54). About half had a bachelor’s degree (49/99, 50%) and less than 3 years of work experience (48/99, 49%). All of them completed the IMMS and knowledge tests. Among those nurses who undertook the education, 84% (83/99) of them (25 ENs and 58 RNs) completed the questionnaire on their perceived training transfer at workplace.

### Motivational Reaction

As shown in Table 1, the overall IMMS mean scores (mean 3.78, SD 0.56) of the nurses indicated that they were motivated to learn the e-RAPIDS. The subscale score of the IMMS indicated that the nurses perceived highly positively the practical relevance of the content and were highly satisfied with the program. The program was also perceived to be more stimulating in capturing attention (mean 4.06, SD 0.52 vs mean 3.00, SD 0.48, *P*<.001) as well as building a higher level of confidence (mean 3.83, SD 0.44 vs mean 2.73, SD 0.53, *P*<.001) among the RNs than the ENs. The overall scores of the RNs (mean 4.02, SD 0.43) were significantly higher (*P*<.001) than the ENs (mean 3.34, SD 0.51).

**Table 1:** Mean motivation scores (N=99).

		Mean (SD)	95% confidence interval
Overall		3.78 (0.56)	3.67-3.89
**Subscales**			
	Attention	3.67 (0.73)	3.52-3.81
	Relevance	4.11 (0.58)	3.99-4.22
	Confidence	3.44 (0.71)	3.30-3.58
	Satisfaction	4.01 (0.71)	3.87-4.15

### Knowledge

After the educational intervention, the RN group demonstrated a significant increase (*P*<.001) on knowledge posttest scores (mean 22.47, SD 2.99) from pretest score (mean 18.80, SD 3.05). Similarly, the EN group also showed a significant improvement (*P*<.001) on the knowledge posttest scores (mean 19.57, SD 3.97) from the pretest score (mean 16.57, SD 3.99).

### Training Transfer at Workplace

As shown in Table 2, the participants demonstrated positive attitudes (mean 3.89, SD 0.49) toward the transfer of learning to clinical practice with mean scores on each item that ranged from 3.39-4.13. No significant difference was found between the RNs (mean 3.82, SD 0.52) and ENs (mean 4.06, SD 0.39).

**Table 2:** Perceived attitudes towards training transfer among the nurses (N=83).

Items	Mean (SD)	95% confidence interval
I will make a plan to put into practice what I have learned after I get back to the workplace.	3.99 (0.57)	3.86-4.11
I will work as hard as possible to put into practice what I have learned for the patients’ benefit.	4.05 (0.62)	3.91-4.18
My work is more organized after I have put into practice what I have learned from the training.	3.87 (0.60)	3.74-4.00
It will be disgraceful if I do not put into practice what I have learned from the training I attended.	3.72 (0.77)	3.55-3.89
I am sure that what I have learned from the training is put into practice for the patients’ benefit.	4.13 (0.54)	4.02-4.25
I feel motivated toward my role in patient deterioration after having attended the training programs.	3.95 (0.71)	3.80-4.11
My commitment towards my role in patient deterioration has increased as a result of attending the training programs.	3.90 (0.76)	3.74-4.07
Supervisors or peers have told me that my performance has improved following the training programs.	3.39 (0.68)	3.24-3.53
I work with more confidence after putting into practice what I have learned from the training.	3.95 (0.64)	3.81-4.09
I have changed my behavior in order to be consistent with the material taught in the training programs.	3.87 (0.62)	3.73-4.00
I knew that I would benefit from the training.	4.07 (0.69)	3.92-4.22
My work performance improved after I attended the training.	3.88 (0.65)	3.74-4.02
My work will be rewarded if I put into practice what I have learned.	3.75 (0.66)	3.60-3.89
I am capable of putting into practice what I have learned from the training even though I am busy.	3.98 (0.54)	3.86-4.09

### Trigger Cases

**Figure 2:**
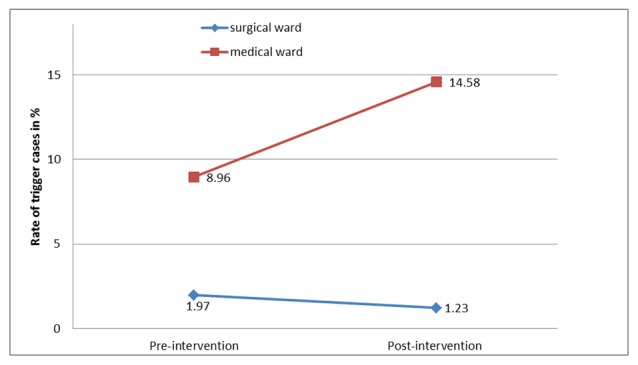
Rate of trigger cases at 6 months pre-intervention and 6 months postintervention.

We studied 2155 patients during the pre-intervention period compared to 1841 patients during the postintervention period. The characteristics were similar between the two periods, but there was a significant increase (*P<*.05) in hospital length of stay in the surgical unit (see Table 3). As shown in Figure 2, the number of cases triggered by nurses in the medical ward increased significantly (*P*<.001) from 8.96% (84/937) in the pre-intervention period to 14.58% (91/624) in the postintervention period. However, no significant difference (*P=*.15) between the pre- (24/1218, 1.97%) and postintervention period (15/1217, 1.23%) was found in the surgical unit. The analysis of each type of trigger did not show any significant differences between the pre- and postintervention periods.

**Table 3:** Characteristics of the two wards during the study period.

		6 months, pre- (n=2155)	6 months, post (n=1841)	*P* value
**Age of patients in years, mean (SD)**				
	Surgical unit	60.64 (17.78)	61.11 (18.01)	.21
	Medical unit	68.30 (18.35)	69.73 (18.96)	.51
**Length of stay in days, mean (SD)**				
	Surgical unit	5.68 (7.71)	6.01 (9.36)	.02
	Medical unit	6.50 (7.54)	6.66 (8.27)	.06
**Occupied beds per day, mean (SD)**				
	Surgical unit	42.29 (0.79)	41.79 (0.87)	.21
	Medical unit	43.10 (0.63)	43.11 (0.32)	.84

## Discussion

### Principal Findings

We measured the outcomes across the levels of an existing adaptation of Kirpatrick’s model [22] during a 14-month period to evaluate the impact of e-RAPIDS. At Level 4a, change in organization practice, we found a significantly increased number of cases being triggered by nurses in the medical ward but no changes in the surgical ward. This could be due to the characteristics of patients admitted to the medical ward who were more likely to deteriorate from their underlying medical diagnosis and comorbidities than the surgical patients. Nevertheless, the improved outcome from the triggering data of the medical ward provided some evidence to support the effectiveness of e-RAPIDS in improving nursing practice on recognizing and responding to deteriorating ward patients. This evidence was further supported by the self-reported perceived training transfer (Level 3) that verified the change of nurses’ behaviors in their workplace after the educational intervention. This improvement in nursing practice could ultimately lead to better patient outcomes. However, as the intervention focused only on the afferent limb of RRS, the evaluation of patient outcomes (Level 4b) that are also dependent on the effectiveness of the response team (efferent limb) is beyond the scope of this study.

As shown in our study, the knowledge gained from e-RAPIDS has resulted in the transfer of learning to the nurses’ clinical practice. Consistent with a previous study [20], our findings reinforced the effectiveness of e-RAPIDS in improving the nurses’ knowledge in recognizing and responding to deteriorating ward patients. This study therefore supports the acquisition of this relevant learning content in supporting the role of nurses in their care of deterioriating patients in clinical practice. However, given the relatively low level of confidence reported by the ENs, the level of difficulty of the learning content for the EN version may need further examination. Apart from the learning content, the knowledge gained observed could be uniquely attributed to the variety of instructional strategies incorporated into e-RAPIDS. The acquisition of factual knowledge using multimedia such as animation video, text, and audio was followed by the application of knowledge through repetition, practice, and feedback in the virtual patient scenarios [19]. The resemblance of the virtual patient scenarios to equivalent real-life scenarios provides an authentic learning context that reflects the way knowledge and skills will be applied in the actual workplace [18]. This authenticity was identified as a critical determinant of learning transfer [26].

Nurses’ positive motivation to engage in e-RAPIDS was reported in this study, demonstrating the acceptability of this learning strategy as part of their continuing nursing education. Similar to the learning of cardiopulmonary resuscitation, the opportunity to engage in repetitive training is essential for the retention of knowledge and skills in assessing and managing deteriorating ward patients. A previous study demonstrated the effective use of either the virtual patient simulation or mannequin-based simulation, with no superiority over each other, as a refresher learning strategy for maintenance of clinical performances [18]. However, with the high resource intensity associated with the mannequin-based simulation, the feasibility of engaging in repetitive training to maintain the competency of a large group of hospital nurses is challenging [19]. In contrast, e-RAPIDS provides unlimited training opportunities, which makes it a viable option for training a large number of nurses to achieve long-term retention of learning. Apart from using it as a refresher training course, e-RAPIDS could serve as a promising self-directed learning strategy to prepare nurses for mannequin-based simulation experience [19]. Future training could implement these learning strategies, forming part of a blended-learning strategy [27], to optimize the impact of RRS. While the e-RAPIDS could emphasize the nursing role in assessing and managing a deteriorating patient, the mannequin-based simulation could extend this to involve effective communication and teamwork through interprofessional training. Future studies could evaluate the effectiveness of this blended-learning strategy on the RRS by evaluating patient outcomes.

### Limitations

An important limitation of this study is the lack of a control group that received no educational intervention. The vast difference in characteristics between the wards at the study hospital made it difficult to identify the control wards that are comparable to the intervention wards. Nevertheless, the before-and-after design, the follow-up at 2-3 months after training, and the triangulation of the results provided reassurance on the robustness of the study outcomes. Another limitation is the clinical outcome measure that we evaluated based on the documented triggers. Given the short timeline of this study, the triggering rates were measured for a relatively short period. Moreover, due to the logistic constraints of this study, we did not assess triggers that were missed from the vital signs documentation.

### Conclusion

Using a before-and-after study design that was conducted over a 14-month period, this study provides evidence on the effectiveness of e-RAPIDS on improving nursing practice in recognizing and responding to deteriorating patients. This evidence was demonstrated by the significantly increased number of deteriorating patients triggered by ward nurses in the medical ward after the implementation of the educational intervention. The study also provides evidence on the knowledge gained from e-RAPIDS that resulted in the transfer of learning to the nurses’ clinical practice. This Web-based simulation could be used by nurse educators worldwide to address the educational needs of nurses in clinical deterioration. Future effort is needed to optimize the use of e-RAPIDS with other educational strategies such as interprofessional simulation training and to evaluate the impact of this blended-learning strategy on patient outcomes.

## Abbreviations

ABDCDE: airway, breathing, circulation, disability, and expose/examine
ENs: enrolled nurses
EWSS: Early Warning Score System
IMMS: Instructional Material Motivation Survey
ISBAR: identity, situation, background, assessment, and recommendation
MCQ: multiple choice questionnaire
NUH: National University Hospital
NUS: National University of Singapore
RAPIDS: Rescuing A Patient In Deteriorating Situations
RCT: randomized controlled trial
RN: registered nurse
RRS: Rapid Response System
